# Supplementary material for: Mechanistic insight into the role of AUXIN RESISTANCE4 in trafficking of AUXIN1 and LIKE AUX1-2
Source: Plant Physiol. 2023 Sep 30;194(1):422–33. doi: 10.1093/plphys/kiad506 (PMC10756756; doi:10.1093/plphys/kiad506)
Supplement: kiad506_Supplementary_Data [file kiad506_supplementary_data.zip › Suppemental Dataset S1/5445e4f6eaa2385e-AtAXR4/summary.html]

Phyre 2 Results for AtAXR4\_\_\_


|  |  |
| --- | --- |
|  | Retrieve Phyre Job Id |


|  |  |
| --- | --- |
| Email | charlie.hodgman@nottinghamac.uk |
| Description | AtAXR4\_\_\_ |
| Date | Tue Jan 10 14:09:39 GMT 2023 |
| Unique Job ID | 5445e4f6eaa2385e |
| Sequence | MAIITEEEED ... Download FASTA |
| Job Type | **normal** |
| Job Expiry |  |

|  |  |
| --- | --- |
| Summary |  |

Top model

Image coloured by rainbow N → C terminus

|  |  |
| --- | --- |
| Model dimensions (Å): | **X**:45.600 **Y**:54.531 **Z**:52.720 |

|  |  |
| --- | --- |
|  |  |
 Close JSmol | | |

 Model (left) based on template c6g75B\_ Top template information **PDB header:**hydrolase **Chain:** B: **PDB Molecule:**common ancestor of haloalkane dehalogenase and renilla **PDBTitle:** crystal structure of the common ancestor of haloalkane dehalogenases2 and renilla luciferase (anchld-rluc) **PDB Entry:** PDBe RCSB PDBj - Confidence and coverage Confidence: \| **100.0%** \| Coverage: \| **57%** 268 residues ( 57% of your sequence) have been modelled with 100.0% confidence by the single highest scoring template. You may wish to submit your sequence to Phyrealarm. This will automatically scan your sequence every week for new potential templates as they appear in the Phyre2 library. 3D viewing Interactive 3D view in JSmol For other options to view your downloaded structure offline see the FAQ |

|  |  |
| --- | --- |
| Sequence analysis |  |

|  |  |
| --- | --- |
|  | Download FASTA version |

|  |  |
| --- | --- |
| Secondary structure and disorder prediction [Show] |  |

|  |  |  |  |
| --- | --- | --- | --- |
|  |  |  |  |

|  |  |  |  |  |  |  |  |  |  |  |  |  |  |  |  |  |  |  |  |  |  |  |  |  |  |  |  |  |  |  |  |  |  |  |  |  |  |  |  |  |  |  |  |  |  |  |  |  |  |  |  |  |  |  |  |  |  |  |  |  |  |  |  |  |  |  |  |  |  |  |  |  |  |  |  |  |  |  |  |  |
| --- | --- | --- | --- | --- | --- | --- | --- | --- | --- | --- | --- | --- | --- | --- | --- | --- | --- | --- | --- | --- | --- | --- | --- | --- | --- | --- | --- | --- | --- | --- | --- | --- | --- | --- | --- | --- | --- | --- | --- | --- | --- | --- | --- | --- | --- | --- | --- | --- | --- | --- | --- | --- | --- | --- | --- | --- | --- | --- | --- | --- | --- | --- | --- | --- | --- | --- | --- | --- | --- | --- | --- | --- | --- | --- | --- | --- | --- | --- | --- | --- |
|  |  | 1 | . | . | . | . | . | . | . | . | 10 | . | . | . | . | . | . | . | . | . | 20 | . | . | . | . | . | . | . | . | . | 30 | . | . | . | . | . | . | . | . | . | 40 | . | . | . | . | . | . | . | . | . | 50 | . | . | . | . | . | . | . | . | . | 60 |  |  |  |  |  |  |  |  |  |  |  |  |  |  |  |  |  |  |  |
| Sequence |  | M | A | I | I | T | E | E | E | E | D | P | K | T | L | N | P | P | K | N | K | P | K | D | S | D | F | T | K | S | E | S | T | M | K | N | P | K | P | Q | S | Q | N | P | F | P | F | W | F | Y | F | T | V | V | V | S | L | A | T | I | I |  |  |  |  |  |  |  |  |  |  |  |  |  |  |  |  |  |  |  |
| Secondary structure |  | --- | --- | --- | --- | --- | --- | --- | --- | --- | --- | --- | --- | --- | --- | --- | --- | --- | --- | --- | --- | --- | --- |  |  |  |  |  |  |  |  |  |  |  |  | --- | --- | --- | --- | --- | --- | --- | --- | --- | --- |  |  |  |  |  |  |  |  |  |  |  |  |  |  |  |  |  |  |  |  |  |  |  |  |  |  |  |  |  |  |  |  |  |  |  |
| SS confidence |  | --- | --- | --- | --- | --- | --- | --- | --- | --- | --- | --- | --- | --- | --- | --- | --- | --- | --- | --- | --- | --- | --- | --- | --- | --- | --- | --- | --- | --- | --- | --- | --- | --- | --- | --- | --- | --- | --- | --- | --- | --- | --- | --- | --- | --- | --- | --- | --- | --- | --- | --- | --- | --- | --- | --- | --- | --- | --- | --- | --- |  |  |  |  |  |  |  |  |  |  |  |  |  |  |  |  |  |  |  |
| Disorder |  | **?** | **?** | **?** | **?** | **?** | **?** | **?** | --- | **?** | **?** | **?** | **?** | **?** | **?** | **?** | **?** | **?** | **?** | **?** | **?** | **?** | **?** | **?** | **?** | **?** | --- | **?** | **?** | **?** | --- | --- | --- | --- | --- | **?** | **?** | --- | --- | --- | --- | --- | --- | --- | --- | --- | --- | --- | --- | --- | --- | --- | --- | --- | --- | --- | --- | --- | --- | --- | --- |  |  |  |  |  |  |  |  |  |  |  |  |  |  |  |  |  |  |  |
| Disorder confidence |  | --- | --- | --- | --- | --- | --- | --- | --- | --- | --- | --- | --- | --- | --- | --- | --- | --- | --- | --- | --- | --- | --- | --- | --- | --- | --- | --- | --- | --- | --- | --- | --- | --- | --- | --- | --- | --- | --- | --- | --- | --- | --- | --- | --- | --- | --- | --- | --- | --- | --- | --- | --- | --- | --- | --- | --- | --- | --- | --- | --- |  |  |  |  |  |  |  |  |  |  |  |  |  |  |  |  |  |  |  |
|  | | | | | | | | | | | | | | | | | | | | | | | | | | | | | | | | | | | | | | | | | | | | | | | | | | | | | | | | | | | | | | | | | | | | | | | | | | | | | | | | |
|  |  | . | . | . | . | . | . | . | . | . | 70 | . | . | . | . | . | . | . | . | . | 80 | . | . | . | . | . | . | . | . | . | 90 | . | . | . | . | . | . | . | . | . | 100 | . | . | . | . | . | . | . | . | . | 110 | . | . | . | . | . | . | . | . | . | 120 |  |  |  |  |  |  |  |  |  |  |  |  |  |  |  |  |  |  |  |
| Sequence |  | F | I | S | L | S | L | F | S | S | Q | N | D | P | R | S | W | F | L | S | L | P | P | A | L | R | Q | H | Y | S | N | G | R | T | I | K | V | Q | V | N | S | N | E | S | P | I | E | V | F | V | A | E | S | G | S | I | H | T | E | T | V |  |  |  |  |  |  |  |  |  |  |  |  |  |  |  |  |  |  |  |
| Secondary structure |  |  |  |  |  |  |  | --- | --- | --- | --- | --- | --- | --- | --- |  |  |  |  |  | --- | --- |  |  |  |  |  |  | --- | --- | --- | --- | --- |  |  |  |  |  | --- | --- | --- | --- | --- | --- | --- |  |  |  |  |  |  |  | --- | --- | --- | --- | --- | --- | --- |  |  |  |  |  |  |  |  |  |  |  |  |  |  |  |  |  |  |  |  |  |
| SS confidence |  | --- | --- | --- | --- | --- | --- | --- | --- | --- | --- | --- | --- | --- | --- | --- | --- | --- | --- | --- | --- | --- | --- | --- | --- | --- | --- | --- | --- | --- | --- | --- | --- | --- | --- | --- | --- | --- | --- | --- | --- | --- | --- | --- | --- | --- | --- | --- | --- | --- | --- | --- | --- | --- | --- | --- | --- | --- | --- | --- | --- |  |  |  |  |  |  |  |  |  |  |  |  |  |  |  |  |  |  |  |
| Disorder |  | --- | --- | --- | --- | **?** | **?** | **?** | **?** | **?** | --- | --- | --- | **?** | **?** | **?** | --- | --- | --- | **?** | --- | --- | --- | **?** | **?** | **?** | **?** | --- | --- | --- | --- | **?** | --- | --- | --- | --- | --- | --- | --- | **?** | --- | --- | --- | --- | --- | --- | --- | --- | --- | --- | --- | --- | --- | --- | --- | --- | --- | --- | --- | --- | --- |  |  |  |  |  |  |  |  |  |  |  |  |  |  |  |  |  |  |  |
| Disorder confidence |  | --- | --- | --- | --- | --- | --- | --- | --- | --- | --- | --- | --- | --- | --- | --- | --- | --- | --- | --- | --- | --- | --- | --- | --- | --- | --- | --- | --- | --- | --- | --- | --- | --- | --- | --- | --- | --- | --- | --- | --- | --- | --- | --- | --- | --- | --- | --- | --- | --- | --- | --- | --- | --- | --- | --- | --- | --- | --- | --- | --- |  |  |  |  |  |  |  |  |  |  |  |  |  |  |  |  |  |  |  |
|  | | | | | | | | | | | | | | | | | | | | | | | | | | | | | | | | | | | | | | | | | | | | | | | | | | | | | | | | | | | | | | | | | | | | | | | | | | | | | | | | |
|  |  | . | . | . | . | . | . | . | . | . | 130 | . | . | . | . | . | . | . | . | . | 140 | . | . | . | . | . | . | . | . | . | 150 | . | . | . | . | . | . | . | . | . | 160 | . | . | . | . | . | . | . | . | . | 170 | . | . | . | . | . | . | . | . | . | 180 |  |  |  |  |  |  |  |  |  |  |  |  |  |  |  |  |  |  |  |
| Sequence |  | V | I | V | H | G | L | G | L | S | S | F | A | F | K | E | M | I | Q | S | L | G | S | K | G | I | H | S | V | A | I | D | L | P | G | N | G | F | S | D | K | S | M | V | V | I | G | G | D | R | E | I | G | F | V | A | R | V | K | E | V |  |  |  |  |  |  |  |  |  |  |  |  |  |  |  |  |  |  |  |
| Secondary structure |  |  |  |  | --- | --- | --- | --- | --- | --- |  |  |  |  |  |  |  |  |  |  |  |  |  | --- | --- | --- |  |  |  |  |  | --- | --- | --- | --- | --- | --- | --- | --- | --- | --- | --- | --- | --- | --- | --- | --- | --- | --- | --- | --- | --- | --- |  |  |  |  |  |  |  |  |  |  |  |  |  |  |  |  |  |  |  |  |  |  |  |  |  |  |  |
| SS confidence |  | --- | --- | --- | --- | --- | --- | --- | --- | --- | --- | --- | --- | --- | --- | --- | --- | --- | --- | --- | --- | --- | --- | --- | --- | --- | --- | --- | --- | --- | --- | --- | --- | --- | --- | --- | --- | --- | --- | --- | --- | --- | --- | --- | --- | --- | --- | --- | --- | --- | --- | --- | --- | --- | --- | --- | --- | --- | --- | --- | --- |  |  |  |  |  |  |  |  |  |  |  |  |  |  |  |  |  |  |  |
| Disorder |  | --- | --- | --- | --- | --- | --- | --- | --- | --- | --- | --- | --- | --- | --- | --- | --- | --- | --- | --- | --- | --- | --- | --- | --- | --- | --- | --- | --- | --- | --- | --- | --- | --- | --- | --- | --- | --- | --- | --- | --- | **?** | **?** | **?** | **?** | **?** | **?** | --- | --- | --- | --- | --- | --- | --- | --- | --- | --- | --- | --- | --- | --- |  |  |  |  |  |  |  |  |  |  |  |  |  |  |  |  |  |  |  |
| Disorder confidence |  | --- | --- | --- | --- | --- | --- | --- | --- | --- | --- | --- | --- | --- | --- | --- | --- | --- | --- | --- | --- | --- | --- | --- | --- | --- | --- | --- | --- | --- | --- | --- | --- | --- | --- | --- | --- | --- | --- | --- | --- | --- | --- | --- | --- | --- | --- | --- | --- | --- | --- | --- | --- | --- | --- | --- | --- | --- | --- | --- | --- |  |  |  |  |  |  |  |  |  |  |  |  |  |  |  |  |  |  |  |
|  | | | | | | | | | | | | | | | | | | | | | | | | | | | | | | | | | | | | | | | | | | | | | | | | | | | | | | | | | | | | | | | | | | | | | | | | | | | | | | | | |
|  |  | . | . | . | . | . | . | . | . | . | 190 | . | . | . | . | . | . | . | . | . | 200 | . | . | . | . | . | . | . | . | . | 210 | . | . | . | . | . | . | . | . | . | 220 | . | . | . | . | . | . | . | . | . | 230 | . | . | . | . | . | . | . | . | . | 240 |  |  |  |  |  |  |  |  |  |  |  |  |  |  |  |  |  |  |  |
| Sequence |  | Y | G | L | I | Q | E | K | G | V | F | W | A | F | D | Q | M | I | E | T | G | D | L | P | Y | E | E | I | I | K | L | Q | N | S | K | R | R | S | F | K | A | I | E | L | G | S | E | E | T | A | R | V | L | G | Q | V | I | D | T | L | G |  |  |  |  |  |  |  |  |  |  |  |  |  |  |  |  |  |  |  |
| Secondary structure |  |  |  |  |  |  |  | --- | --- | --- |  |  |  |  |  |  |  |  |  |  |  |  |  |  |  |  |  |  |  |  | --- | --- | --- | --- | --- | --- | --- | --- | --- |  |  |  | --- | --- | --- | --- | --- | --- |  |  |  |  |  |  |  |  |  |  |  | --- | --- |  |  |  |  |  |  |  |  |  |  |  |  |  |  |  |  |  |  |  |
| SS confidence |  | --- | --- | --- | --- | --- | --- | --- | --- | --- | --- | --- | --- | --- | --- | --- | --- | --- | --- | --- | --- | --- | --- | --- | --- | --- | --- | --- | --- | --- | --- | --- | --- | --- | --- | --- | --- | --- | --- | --- | --- | --- | --- | --- | --- | --- | --- | --- | --- | --- | --- | --- | --- | --- | --- | --- | --- | --- | --- | --- | --- |  |  |  |  |  |  |  |  |  |  |  |  |  |  |  |  |  |  |  |
| Disorder |  | --- | --- | --- | --- | --- | --- | --- | --- | --- | --- | --- | --- | --- | --- | --- | --- | --- | --- | --- | --- | --- | --- | --- | --- | --- | --- | --- | --- | --- | --- | **?** | **?** | **?** | **?** | **?** | **?** | **?** | --- | --- | --- | --- | --- | --- | --- | --- | --- | **?** | --- | --- | --- | --- | --- | --- | --- | --- | --- | --- | --- | --- | --- |  |  |  |  |  |  |  |  |  |  |  |  |  |  |  |  |  |  |  |
| Disorder confidence |  | --- | --- | --- | --- | --- | --- | --- | --- | --- | --- | --- | --- | --- | --- | --- | --- | --- | --- | --- | --- | --- | --- | --- | --- | --- | --- | --- | --- | --- | --- | --- | --- | --- | --- | --- | --- | --- | --- | --- | --- | --- | --- | --- | --- | --- | --- | --- | --- | --- | --- | --- | --- | --- | --- | --- | --- | --- | --- | --- | --- |  |  |  |  |  |  |  |  |  |  |  |  |  |  |  |  |  |  |  |
|  | | | | | | | | | | | | | | | | | | | | | | | | | | | | | | | | | | | | | | | | | | | | | | | | | | | | | | | | | | | | | | | | | | | | | | | | | | | | | | | | |
|  |  | . | . | . | . | . | . | . | . | . | 250 | . | . | . | . | . | . | . | . | . | 260 | . | . | . | . | . | . | . | . | . | 270 | . | . | . | . | . | . | . | . | . | 280 | . | . | . | . | . | . | . | . | . | 290 | . | . | . | . | . | . | . | . | . | 300 |  |  |  |  |  |  |  |  |  |  |  |  |  |  |  |  |  |  |  |
| Sequence |  | L | A | P | V | H | L | V | L | H | D | S | A | L | G | L | A | S | N | W | V | S | E | N | W | Q | S | V | R | S | V | T | L | I | D | S | S | I | S | P | A | L | P | L | W | V | L | N | V | P | G | I | R | E | I | L | L | A | F | S | F |  |  |  |  |  |  |  |  |  |  |  |  |  |  |  |  |  |  |  |
| Secondary structure |  | --- | --- | --- | --- |  |  |  |  |  |  |  |  |  |  |  |  |  |  |  |  |  |  |  |  |  |  |  | --- | --- |  |  |  |  | --- | --- | --- | --- | --- |  |  |  |  |  |  |  |  |  | --- | --- | --- |  |  |  |  |  |  |  |  |  |  |  |  |  |  |  |  |  |  |  |  |  |  |  |  |  |  |  |  |  |
| SS confidence |  | --- | --- | --- | --- | --- | --- | --- | --- | --- | --- | --- | --- | --- | --- | --- | --- | --- | --- | --- | --- | --- | --- | --- | --- | --- | --- | --- | --- | --- | --- | --- | --- | --- | --- | --- | --- | --- | --- | --- | --- | --- | --- | --- | --- | --- | --- | --- | --- | --- | --- | --- | --- | --- | --- | --- | --- | --- | --- | --- | --- |  |  |  |  |  |  |  |  |  |  |  |  |  |  |  |  |  |  |  |
| Disorder |  | --- | --- | --- | --- | --- | --- | --- | --- | --- | --- | --- | --- | --- | --- | --- | --- | --- | --- | --- | --- | --- | --- | --- | --- | --- | --- | **?** | --- | --- | --- | --- | --- | **?** | **?** | --- | **?** | **?** | **?** | --- | --- | --- | --- | --- | --- | --- | --- | --- | --- | --- | --- | --- | --- | --- | --- | --- | **?** | **?** | **?** | --- | **?** |  |  |  |  |  |  |  |  |  |  |  |  |  |  |  |  |  |  |  |
| Disorder confidence |  | --- | --- | --- | --- | --- | --- | --- | --- | --- | --- | --- | --- | --- | --- | --- | --- | --- | --- | --- | --- | --- | --- | --- | --- | --- | --- | --- | --- | --- | --- | --- | --- | --- | --- | --- | --- | --- | --- | --- | --- | --- | --- | --- | --- | --- | --- | --- | --- | --- | --- | --- | --- | --- | --- | --- | --- | --- | --- | --- | --- |  |  |  |  |  |  |  |  |  |  |  |  |  |  |  |  |  |  |  |
|  | | | | | | | | | | | | | | | | | | | | | | | | | | | | | | | | | | | | | | | | | | | | | | | | | | | | | | | | | | | | | | | | | | | | | | | | | | | | | | | | |
|  |  | . | . | . | . | . | . | . | . | . | 310 | . | . | . | . | . | . | . | . | . | 320 | . | . | . | . | . | . | . | . | . | 330 | . | . | . | . | . | . | . | . | . | 340 | . | . | . | . | . | . | . | . | . | 350 | . | . | . | . | . | . | . | . | . | 360 |  |  |  |  |  |  |  |  |  |  |  |  |  |  |  |  |  |  |  |
| Sequence |  | G | F | E | K | L | V | S | F | R | C | S | K | E | M | T | L | S | D | I | D | A | H | R | I | L | L | K | G | R | N | G | R | E | A | V | V | A | S | L | N | K | L | N | H | S | F | D | I | A | Q | W | G | N | S | D | G | I | N | G | I |  |  |  |  |  |  |  |  |  |  |  |  |  |  |  |  |  |  |  |
| Secondary structure |  |  |  |  |  |  |  |  |  |  | --- | --- | --- | --- | --- | --- |  |  |  |  |  |  |  |  |  |  |  |  | --- | --- | --- |  |  |  |  |  |  |  |  |  |  |  | --- | --- | --- | --- | --- |  |  |  |  |  |  |  | --- | --- | --- | --- | --- | --- | --- |  |  |  |  |  |  |  |  |  |  |  |  |  |  |  |  |  |  |  |
| SS confidence |  | --- | --- | --- | --- | --- | --- | --- | --- | --- | --- | --- | --- | --- | --- | --- | --- | --- | --- | --- | --- | --- | --- | --- | --- | --- | --- | --- | --- | --- | --- | --- | --- | --- | --- | --- | --- | --- | --- | --- | --- | --- | --- | --- | --- | --- | --- | --- | --- | --- | --- | --- | --- | --- | --- | --- | --- | --- | --- | --- | --- |  |  |  |  |  |  |  |  |  |  |  |  |  |  |  |  |  |  |  |
| Disorder |  | --- | --- | --- | --- | --- | --- | --- | --- | **?** | **?** | **?** | **?** | --- | --- | --- | --- | --- | --- | --- | --- | --- | --- | --- | --- | --- | --- | --- | --- | --- | --- | --- | --- | --- | --- | --- | --- | --- | --- | --- | --- | --- | --- | --- | **?** | **?** | **?** | --- | **?** | **?** | --- | **?** | **?** | **?** | --- | **?** | **?** | **?** | --- | --- | --- |  |  |  |  |  |  |  |  |  |  |  |  |  |  |  |  |  |  |  |
| Disorder confidence |  | --- | --- | --- | --- | --- | --- | --- | --- | --- | --- | --- | --- | --- | --- | --- | --- | --- | --- | --- | --- | --- | --- | --- | --- | --- | --- | --- | --- | --- | --- | --- | --- | --- | --- | --- | --- | --- | --- | --- | --- | --- | --- | --- | --- | --- | --- | --- | --- | --- | --- | --- | --- | --- | --- | --- | --- | --- | --- | --- | --- |  |  |  |  |  |  |  |  |  |  |  |  |  |  |  |  |  |  |  |
|  | | | | | | | | | | | | | | | | | | | | | | | | | | | | | | | | | | | | | | | | | | | | | | | | | | | | | | | | | | | | | | | | | | | | | | | | | | | | | | | | |
|  |  | . | . | . | . | . | . | . | . | . | 370 | . | . | . | . | . | . | . | . | . | 380 | . | . | . | . | . | . | . | . | . | 390 | . | . | . | . | . | . | . | . | . | 400 | . | . | . | . | . | . | . | . | . | 410 | . | . | . | . | . | . | . | . | . | 420 |  |  |  |  |  |  |  |  |  |  |  |  |  |  |  |  |  |  |  |
| Sequence |  | P | M | Q | V | I | W | S | S | E | A | S | K | E | W | S | D | E | G | Q | R | V | A | K | A | L | P | K | A | K | F | V | T | H | S | G | S | R | W | P | Q | E | S | K | S | G | E | L | A | D | Y | I | S | E | F | V | S | L | L | P | K |  |  |  |  |  |  |  |  |  |  |  |  |  |  |  |  |  |  |  |
| Secondary structure |  | --- |  |  |  |  |  |  | --- | --- | --- | --- | --- | --- | --- |  |  |  |  |  |  |  |  |  |  | --- | --- | --- | --- |  |  |  |  |  | --- | --- | --- | --- | --- | --- | --- | --- | --- | --- |  |  |  |  |  |  |  |  |  |  |  |  |  |  | --- | --- | --- |  |  |  |  |  |  |  |  |  |  |  |  |  |  |  |  |  |  |  |
| SS confidence |  | --- | --- | --- | --- | --- | --- | --- | --- | --- | --- | --- | --- | --- | --- | --- | --- | --- | --- | --- | --- | --- | --- | --- | --- | --- | --- | --- | --- | --- | --- | --- | --- | --- | --- | --- | --- | --- | --- | --- | --- | --- | --- | --- | --- | --- | --- | --- | --- | --- | --- | --- | --- | --- | --- | --- | --- | --- | --- | --- | --- |  |  |  |  |  |  |  |  |  |  |  |  |  |  |  |  |  |  |  |
| Disorder |  | --- | --- | --- | --- | --- | --- | --- | --- | --- | --- | --- | --- | --- | --- | --- | --- | --- | --- | --- | --- | --- | --- | --- | --- | --- | --- | --- | --- | --- | --- | --- | --- | --- | --- | --- | --- | --- | --- | --- | --- | --- | --- | --- | --- | --- | --- | --- | --- | --- | --- | --- | --- | --- | --- | --- | --- | --- | **?** | --- | **?** |  |  |  |  |  |  |  |  |  |  |  |  |  |  |  |  |  |  |  |
| Disorder confidence |  | --- | --- | --- | --- | --- | --- | --- | --- | --- | --- | --- | --- | --- | --- | --- | --- | --- | --- | --- | --- | --- | --- | --- | --- | --- | --- | --- | --- | --- | --- | --- | --- | --- | --- | --- | --- | --- | --- | --- | --- | --- | --- | --- | --- | --- | --- | --- | --- | --- | --- | --- | --- | --- | --- | --- | --- | --- | --- | --- | --- |  |  |  |  |  |  |  |  |  |  |  |  |  |  |  |  |  |  |  |
|  | | | | | | | | | | | | | | | | | | | | | | | | | | | | | | | | | | | | | | | | | | | | | | | | | | | | | | | | | | | | | | | | | | | | | | | | | | | | | | | | |
|  |  | . | . | . | . | . | . | . | . | . | 430 | . | . | . | . | . | . | . | . | . | 440 | . | . | . | . | . | . | . | . | . | 450 | . | . | . | . | . | . | . | . | . | 460 | . | . | . | . | . | . | . | . | . | 470 | . | . | . |  |  |  |  |  |  |  |  |  |  |  |  |  |  |  |  |  |  |  |  |  |  |  |  |  |  |
| Sequence |  | S | I | R | R | V | A | E | E | P | I | P | E | E | V | Q | K | V | L | E | E | A | K | A | G | D | D | H | D | H | H | H | G | H | G | H | A | H | A | G | Y | S | D | A | Y | G | L | G | E | E | W | T | T | T |  |  |  |  |  |  |  |  |  |  |  |  |  |  |  |  |  |  |  |  |  |  |  |  |  |  |
| Secondary structure |  | --- | --- | --- | --- | --- | --- | --- | --- | --- | --- |  |  |  |  |  |  |  |  |  |  |  |  | --- | --- | --- | --- | --- | --- | --- | --- | --- | --- | --- | --- | --- | --- | --- | --- | --- | --- | --- | --- | --- | --- | --- | --- | --- | --- |  |  |  | --- | --- |  |  |  |  |  |  |  |  |  |  |  |  |  |  |  |  |  |  |  |  |  |  |  |  |  |  |
| SS confidence |  | --- | --- | --- | --- | --- | --- | --- | --- | --- | --- | --- | --- | --- | --- | --- | --- | --- | --- | --- | --- | --- | --- | --- | --- | --- | --- | --- | --- | --- | --- | --- | --- | --- | --- | --- | --- | --- | --- | --- | --- | --- | --- | --- | --- | --- | --- | --- | --- | --- | --- | --- | --- | --- |  |  |  |  |  |  |  |  |  |  |  |  |  |  |  |  |  |  |  |  |  |  |  |  |  |  |
| Disorder |  | **?** | **?** | **?** | **?** | **?** | **?** | **?** | **?** | **?** | **?** | --- | --- | --- | --- | --- | --- | --- | --- | --- | --- | --- | --- | **?** | **?** | **?** | **?** | **?** | **?** | **?** | **?** | **?** | **?** | **?** | **?** | **?** | **?** | **?** | **?** | **?** | **?** | **?** | **?** | **?** | **?** | --- | **?** | **?** | **?** | **?** | **?** | **?** | **?** | **?** |  |  |  |  |  |  |  |  |  |  |  |  |  |  |  |  |  |  |  |  |  |  |  |  |  |  |
| Disorder confidence |  | --- | --- | --- | --- | --- | --- | --- | --- | --- | --- | --- | --- | --- | --- | --- | --- | --- | --- | --- | --- | --- | --- | --- | --- | --- | --- | --- | --- | --- | --- | --- | --- | --- | --- | --- | --- | --- | --- | --- | --- | --- | --- | --- | --- | --- | --- | --- | --- | --- | --- | --- | --- | --- |  |  |  |  |  |  |  |  |  |  |  |  |  |  |  |  |  |  |  |  |  |  |  |  |  |  |
|  | | | | | | | | | | | | | | | | | | | | | | | | | | | | | | | | | | | | | | | | | | | | | | | | | | | | | | | | | | | | | | | | | | | | | | | | | | | | | | | | |

|  |  |  |
| --- | --- | --- |
| Confidence Key \| \| \| \| \| \| \| \| \| \| \|; High(9) \| \| \| \| \| \| \| \| \| \| \| Low (0) | **?** \| Disordered ( 26%); \| Alpha helix ( 47%) | Beta strand ( 8%) |
|  | TM helix ( 4%) || (View membrane topology prediction) | |
  |

|  |  |
| --- | --- |
| Domain analysis [Show] |  |

|  |  |  |  |
| --- | --- | --- | --- |
|  |  |  |  |

|  |
| --- |
| (Rank \| Aligned region) |
| (1 \| (--- \| c6g75B\_ \| ---); PDB 6g75 chain B **Region:** 86 - 419 **Aligned:** 268 **Modelled:** 277 **Confidence:** 100.0% **Identity:** 24% **PDB header:**hydrolase **Chain:** B: **PDB Molecule:**common ancestor of haloalkane dehalogenase and renilla **PDBTitle:** crystal structure of the common ancestor of haloalkane dehalogenases2 and renilla luciferase (anchld-rluc) **PDB Entry:** PDBe RCSB PDBj Phyre2; 2 \| (--- \| c2vf2A\_ \| ---); PDB 2vf2 chain A **Region:** 92 - 417 **Aligned:** 266 **Modelled:** 269 **Confidence:** 100.0% **Identity:** 24% **PDB header:**hydrolase **Chain:** A: **PDB Molecule:**2-hydroxy-6-oxo-6-phenylhexa-2,4-dienoate **PDBTitle:** x-ray crystal structure of hsad from mycobacterium2 tuberculosis **PDB Entry:** PDBe RCSB PDBj Phyre2; 3 \| (--- \| c4mj3B\_ \| ---); PDB 4mj3 chain B **Region:** 89 - 419 **Aligned:** 274 **Modelled:** 275 **Confidence:** 100.0% **Identity:** 22% **PDB header:**hydrolase **Chain:** B: **PDB Molecule:**haloalkane dehalogenase; **PDBTitle:** haloalkane dehalogenase dmra from mycobacterium rhodesiae js60 **PDB Entry:** PDBe RCSB PDBj Phyre2; 4 \| (--- \| d1bn7a\_ \| ---); PDB 1bn7 chain A **Region:** 104 - 418 **Aligned:** 256 **Modelled:** 257 **Confidence:** 100.0% **Identity:** 23% **Fold:** alpha/beta-Hydrolases **Superfamily:** alpha/beta-Hydrolases **Family:** Haloalkane dehalogenase Phyre2; 5 \| (--- \| c3i28A\_ \| ---); PDB 3i28 chain A **Region:** 72 - 417 **Aligned:** 283 **Modelled:** 290 **Confidence:** 100.0% **Identity:** 20% **PDB header:**hydrolase **Chain:** A: **PDB Molecule:**epoxide hydrolase 2; **PDBTitle:** crystal structure of soluble epoxide hydrolase **PDB Entry:** PDBe RCSB PDBj Phyre2; 6 \| (--- \| c1cr6A\_ \| ---); PDB 1cr6 chain A **Region:** 73 - 417 **Aligned:** 282 **Modelled:** 289 **Confidence:** 100.0% **Identity:** 20% **PDB header:**hydrolase **Chain:** A: **PDB Molecule:**epoxide hydrolase; **PDBTitle:** crystal structure of murine soluble epoxide hydrolase2 complexed with cpu inhibitor **PDB Entry:** PDBe RCSB PDBj Phyre2; 7 \| (--- \| c6y9gB\_ \| ---); PDB 6y9g chain B **Region:** 104 - 418 **Aligned:** 254 **Modelled:** 257 **Confidence:** 100.0% **Identity:** 22% **PDB header:**hydrolase **Chain:** B: **PDB Molecule:**ancestral haloalkane dehalogenase anchld5; **PDBTitle:** crystal structure of putative ancestral haloalkane dehalogenase2 anchld5 (node 5) **PDB Entry:** PDBe RCSB PDBj Phyre2; 8 \| (--- \| c4ns4A\_ \| ---); PDB 4ns4 chain A **Region:** 86 - 418 **Aligned:** 259 **Modelled:** 276 **Confidence:** 100.0% **Identity:** 21% **PDB header:**hydrolase **Chain:** A: **PDB Molecule:**alpha/beta hydrolase fold protein; **PDBTitle:** crystal structure of cold-active estarase from psychrobacter2 cryohalolentis k5t **PDB Entry:** PDBe RCSB PDBj Phyre2; 9 \| (--- \| d1mj5a\_ \| ---); PDB 1mj5 chain A **Region:** 88 - 421 **Aligned:** 270 **Modelled:** 273 **Confidence:** 100.0% **Identity:** 20% **Fold:** alpha/beta-Hydrolases **Superfamily:** alpha/beta-Hydrolases **Family:** Haloalkane dehalogenase Phyre2; 10 \| (--- \| c3u1tA\_ \| ---); PDB 3u1t chain A **Region:** 104 - 420 **Aligned:** 257 **Modelled:** 258 **Confidence:** 100.0% **Identity:** 21% **PDB header:**hydrolase **Chain:** A: **PDB Molecule:**dmma haloalkane dehalogenase; **PDBTitle:** haloalkane dehalogenase, dmma, of marine microbial origin **PDB Entry:** PDBe RCSB PDBj Phyre2; 11 \| (--- \| c4c6hA\_ \| ---); PDB 4c6h chain A **Region:** 104 - 418 **Aligned:** 257 **Modelled:** 261 **Confidence:** 100.0% **Identity:** 21% **PDB header:**hydrolase **Chain:** A: **PDB Molecule:**haloalkane dehalogenase; **PDBTitle:** haloalkane dehalogenase with 1-hexanol **PDB Entry:** PDBe RCSB PDBj Phyre2; 12 \| (--- \| c3a2nF\_ \| ---); PDB 3a2n chain F **Region:** 92 - 423 **Aligned:** 267 **Modelled:** 274 **Confidence:** 100.0% **Identity:** 20% **PDB header:**hydrolase **Chain:** F: **PDB Molecule:**haloalkane dehalogenase; **PDBTitle:** crystal structure of dbja (wild type type ii p21) **PDB Entry:** PDBe RCSB PDBj Phyre2; 13 \| (--- \| c3wibB\_ \| ---); PDB 3wib chain B **Region:** 89 - 418 **Aligned:** 265 **Modelled:** 272 **Confidence:** 100.0% **Identity:** 20% **PDB header:**hydrolase **Chain:** B: **PDB Molecule:**haloalkane dehalogenase; **PDBTitle:** crystal structure of y109w mutant haloalkane dehalogenase data from2 agrobacterium tumefaciens c58 **PDB Entry:** PDBe RCSB PDBj Phyre2; 14 \| (--- \| c7jqzE\_ \| ---); PDB 7jqz chain E **Region:** 104 - 416 **Aligned:** 252 **Modelled:** 255 **Confidence:** 100.0% **Identity:** 21% **PDB header:**hydrolase **Chain:** E: **PDB Molecule:**alpha/beta hydrolase fold; **PDBTitle:** crystal structure of cfl2 wild-type from burkholderia cenocepacia **PDB Entry:** PDBe RCSB PDBj Phyre2; 15 \| (--- \| d1cr6a2 \| ---); PDB 1cr6 chain A domain 2 **Region:** 92 - 417 **Aligned:** 261 **Modelled:** 270 **Confidence:** 100.0% **Identity:** 20% **Fold:** alpha/beta-Hydrolases **Superfamily:** alpha/beta-Hydrolases **Family:** Epoxide hydrolase Phyre2; 16 \| (--- \| d1zd3a2 \| ---); PDB 1zd3 chain A domain 2 **Region:** 92 - 417 **Aligned:** 261 **Modelled:** 270 **Confidence:** 100.0% **Identity:** 20% **Fold:** alpha/beta-Hydrolases **Superfamily:** alpha/beta-Hydrolases **Family:** Epoxide hydrolase Phyre2; 17 \| (--- \| c2e3jA\_ \| ---); PDB 2e3j chain A **Region:** 104 - 420 **Aligned:** 260 **Modelled:** 261 **Confidence:** 100.0% **Identity:** 20% **PDB header:**hydrolase **Chain:** A: **PDB Molecule:**epoxide hydrolase ephb; **PDBTitle:** the crystal structure of epoxide hydrolase b (rv1938) from2 mycobacterium tuberculosis at 2.1 angstrom **PDB Entry:** PDBe RCSB PDBj Phyre2; 18 \| (--- \| c7avrG\_ \| ---); PDB 7avr chain G **Region:** 68 - 415 **Aligned:** 286 **Modelled:** 292 **Confidence:** 100.0% **Identity:** 18% **PDB header:**hydrolase **Chain:** G: **PDB Molecule:**haloalkane dehalogenase 1; **PDBTitle:** the tetrameric structure of haloalkane dehalogenase dpaa from2 paraglaciecola agarilytica no2 **PDB Entry:** PDBe RCSB PDBj Phyre2; 19 \| (--- \| c2qvbA\_ \| ---); PDB 2qvb chain A **Region:** 104 - 418 **Aligned:** 257 **Modelled:** 257 **Confidence:** 100.0% **Identity:** 20% **PDB header:**hydrolase **Chain:** A: **PDB Molecule:**haloalkane dehalogenase 3; **PDBTitle:** crystal structure of haloalkane dehalogenase rv2579 from mycobacterium2 tuberculosis **PDB Entry:** PDBe RCSB PDBj Phyre2; 20 \| (--- \| c5xmdA\_ \| ---); PDB 5xmd chain A **Region:** 85 - 416 **Aligned:** 269 **Modelled:** 276 **Confidence:** 100.0% **Identity:** 19% **PDB header:**hydrolase **Chain:** A: **PDB Molecule:**epoxide hydrolase a; **PDBTitle:** crystal structure of epoxide hydrolase vreh1 from vigna radiata **PDB Entry:** PDBe RCSB PDBj Phyre2; 21 \| (--- \| --- \| ---); 22 \| (--- \| --- \| ---); 23 \| (--- \| --- \| ---); 24 \| (--- \| --- \| ---); 25 \| (--- \| --- \| ---); 26 \| (--- \| --- \| ---); 27 \| (--- \| --- \| ---); 28 \| (--- \| --- \| ---); 29 \| (--- \| --- \| ---); 30 \| (--- \| --- \| ---); 31 \| (--- \| --- \| ---); 32 \| (--- \| --- \| ---); 33 \| (--- \| --- \| ---); 34 \| (--- \| --- \| ---); 35 \| (--- \| --- \| ---); 36 \| (--- \| --- \| ---); 37 \| (--- \| --- \| ---); 38 \| (--- \| --- \| ---); 39 \| (--- \| --- \| ---); 40 \| (--- \| --- \| ---); 41 \| (--- \| --- \| ---); 42 \| (--- \| --- \| ---); 43 \| (--- \| --- \| ---); 44 \| (--- \| --- \| ---); 45 \| (--- \| --- \| ---); 46 \| (--- \| --- \| ---); 47 \| (--- \| --- \| ---); 48 \| (--- \| --- \| ---); 49 \| (--- \| --- \| ---); 50 \| (--- \| --- \| ---); 51 \| (--- \| --- \| ---); 52 \| (--- \| --- \| ---); 53 \| (--- \| --- \| ---); 54 \| (--- \| --- \| ---); 55 \| (--- \| --- \| ---); 56 \| (--- \| --- \| ---); 57 \| (--- \| --- \| ---); 58 \| (--- \| --- \| ---); 59 \| (--- \| --- \| ---); 60 \| (--- \| --- \| ---); 61 \| (--- \| --- \| ---); 62 \| (--- \| --- \| ---); 63 \| (--- \| --- \| ---); 64 \| (--- \| --- \| ---); 65 \| (--- \| --- \| ---); 66 \| (--- \| --- \| ---); 67 \| (--- \| --- \| ---); 68 \| (--- \| --- \| ---); 69 \| (--- \| --- \| ---); 70 \| (--- \| --- \| ---); 71 \| (--- \| --- \| ---); 72 \| (--- \| --- \| ---); 73 \| (--- \| --- \| ---); 74 \| (--- \| --- \| ---); 75 \| (--- \| --- \| ---); 76 \| (--- \| --- \| ---); 77 \| (--- \| --- \| ---); 78 \| (--- \| --- \| ---); 79 \| (--- \| --- \| ---); 80 \| (--- \| --- \| ---); 81 \| (--- \| --- \| ---); 82 \| (--- \| --- \| ---); 83 \| (--- \| --- \| ---); 84 \| (--- \| --- \| ---); 85 \| (--- \| --- \| ---); 86 \| (--- \| --- \| ---); 87 \| (--- \| --- \| ---); 88 \| (--- \| --- \| ---); 89 \| (--- \| --- \| ---); 90 \| (--- \| --- \| ---); 91 \| (--- \| --- \| ---); 92 \| (--- \| --- \| ---); 93 \| (--- \| --- \| ---); 94 \| (--- \| --- \| ---); 95 \| (--- \| --- \| ---); 96 \| (--- \| --- \| ---); 97 \| (--- \| --- \| ---); 98 \| (--- \| --- \| ---); 99 \| (--- \| --- \| ---); 100 \| (--- \| --- \| ---); 101 \| (--- \| --- \| ---); 102 \| (--- \| --- \| ---); 103 \| (--- \| --- \| ---); 104 \| (--- \| --- \| ---); 105 \| (--- \| --- \| ---); 106 \| (--- \| --- \| ---); 107 \| (--- \| --- \| ---); 108 \| (--- \| --- \| ---); 109 \| (--- \| --- \| ---); 110 \| (--- \| --- \| ---); 111 \| (--- \| --- \| ---); 112 \| (--- \| --- \| ---); 113 \| (--- \| --- \| ---); 114 \| (--- \| --- \| ---); 115 \| (--- \| --- \| ---); 116 \| (--- \| --- \| ---); 117 \| (--- \| --- \| ---); 118 \| (--- \| --- \| ---); 119 \| (--- \| --- \| ---); 120 \| (--- \| --- \| ---); 121 \| (--- \| --- \| ---); 122 \| (--- \| --- \| ---); 123 \| (--- \| --- \| ---); 124 \| (--- \| --- \| ---); 125 \| (--- \| --- \| ---); 126 \| (--- \| --- \| ---); 127 \| (--- \| --- \| ---); 128 \| (--- \| --- \| ---); 129 \| (--- \| --- \| ---); 130 \| (--- \| --- \| ---); 131 \| (--- \| --- \| ---); 132 \| (--- \| --- \| ---); 133 \| (--- \| --- \| ---); 134 \| (--- \| --- \| ---); 135 \| (--- \| --- \| ---); 136 \| (--- \| --- \| ---); 137 \| (--- \| --- \| ---); 138 \| (--- \| --- \| ---); 139 \| (--- \| --- \| ---); 140 \| (--- \| --- \| ---); 141 \| (--- \| --- \| ---); 142 \| (--- \| --- \| ---); 143 \| (--- \| --- \| ---); 144 \| (--- \| --- \| ---); 145 \| (--- \| --- \| ---); 146 \| (--- \| --- \| ---); 147 \| (--- \| --- \| ---); 148 \| (--- \| --- \| ---); 149 \| (--- \| --- \| ---); 150 \| (--- \| --- \| ---); 151 \| (--- \| --- \| ---); 152 \| (--- \| --- \| ---); 153 \| (--- \| --- \| ---); 154 \| (--- \| --- \| ---); 155 \| (--- \| --- \| ---); 156 \| (--- \| --- \| ---); 157 \| (--- \| --- \| ---); 158 \| (--- \| --- \| ---); 159 \| (--- \| --- \| ---); 160 \| (--- \| --- \| ---); 161 \| (--- \| --- \| ---); 162 \| (--- \| --- \| ---); 163 \| (--- \| --- \| ---); 164 \| (--- \| --- \| ---); 165 \| (--- \| --- \| ---); 166 \| (--- \| --- \| ---); 167 \| (--- \| --- \| ---); 168 \| (--- \| --- \| ---); 169 \| (--- \| --- \| ---); 170 \| (--- \| --- \| ---); 171 \| (--- \| --- \| ---); 172 \| (--- \| --- \| ---); 173 \| (--- \| --- \| ---); 174 \| (--- \| --- \| ---); 175 \| (--- \| --- \| ---); 176 \| (--- \| --- \| ---); 177 \| (--- \| --- \| ---); 178 \| (--- \| --- \| ---); 179 \| (--- \| --- \| ---); 180 \| (--- \| --- \| ---); 181 \| (--- \| --- \| ---); 182 \| (--- \| --- \| ---); 183 \| (--- \| --- \| ---); 184 \| (--- \| --- \| ---); 185 \| (--- \| --- \| ---); 186 \| (--- \| --- \| ---); 187 \| (--- \| --- \| ---); 188 \| (--- \| --- \| ---); 189 \| (--- \| --- \| ---); 190 \| (--- \| --- \| ---); 191 \| (--- \| --- \| ---); 192 \| (--- \| --- \| ---); 193 \| (--- \| --- \| ---); 194 \| (--- \| --- \| ---); 195 \| (--- \| --- \| ---); 196 \| (--- \| --- \| ---); 197 \| (--- \| --- \| ---); 198 \| (--- \| --- \| ---); 199 \| (--- \| --- \| ---); 200 \| (--- \| --- \| ---); 201 \| (--- \| --- \| ---); 202 \| (--- \| --- \| ---); 203 \| (--- \| --- \| ---); 204 \| (--- \| --- \| ---); 205 \| (--- \| --- \| ---); 206 \| (--- \| --- \| ---); 207 \| (--- \| --- \| ---); 208 \| (--- \| --- \| ---); 209 \| (--- \| --- \| ---); 210 \| (--- \| --- \| ---); 211 \| (--- \| --- \| ---); 212 \| (--- \| --- \| ---); 213 \| (--- \| --- \| ---); 214 \| (--- \| --- \| ---); 215 \| (--- \| --- \| ---); 216 \| (--- \| --- \| ---); 217 \| (--- \| --- \| ---); 218 \| (--- \| --- \| ---); 219 \| (--- \| --- \| ---); 220 \| (--- \| --- \| ---); 221 \| (--- \| --- \| ---); 222 \| (--- \| --- \| ---); 223 \| (--- \| --- \| ---); 224 \| (--- \| --- \| ---); 225 \| (--- \| --- \| ---); 226 \| (--- \| --- \| ---); 227 \| (--- \| --- \| ---); 228 \| (--- \| --- \| ---); 229 \| (--- \| --- \| ---); 230 \| (--- \| --- \| ---); 231 \| (--- \| --- \| ---); 232 \| (--- \| --- \| ---); 233 \| (--- \| --- \| ---); 234 \| (--- \| --- \| ---); 235 \| (--- \| --- \| ---); 236 \| (--- \| --- \| ---); 237 \| (--- \| --- \| ---); 238 \| (--- \| --- \| ---); 239 \| (--- \| --- \| ---); 240 \| (--- \| --- \| ---); 241 \| (--- \| --- \| ---); 242 \| (--- \| --- \| ---); 243 \| (--- \| --- \| ---); 244 \| (--- \| --- \| ---); 245 \| (--- \| --- \| ---); 246 \| (--- \| --- \| ---); 247 \| (--- \| --- \| ---); 248 \| (--- \| --- \| ---); 249 \| (--- \| --- \| ---); 250 \| (--- \| --- \| ---); 251 \| (--- \| --- \| ---); 252 \| (--- \| --- \| ---); 253 \| (--- \| --- \| ---); 254 \| (--- \| --- \| ---); 255 \| (--- \| --- \| ---); 256 \| (--- \| --- \| ---); 257 \| (--- \| --- \| ---); 258 \| (--- \| --- \| ---); 259 \| (--- \| --- \| ---); 260 \| (--- \| --- \| ---); 261 \| (--- \| --- \| ---); 262 \| (--- \| --- \| ---); 263 \| (--- \| --- \| ---); 264 \| (--- \| --- \| ---); 265 \| (--- \| --- \| ---); 266 \| (--- \| --- \| ---); 267 \| (--- \| --- \| ---); 268 \| (--- \| --- \| ---); 269 \| (--- \| --- \| ---); 270 \| (--- \| --- \| ---); 271 \| (--- \| --- \| ---); 272 \| (--- \| --- \| ---); 273 \| (--- \| --- \| ---); 274 \| (--- \| --- \| ---); 275 \| (--- \| --- \| ---); 276 \| (--- \| --- \| ---); 277 \| (--- \| --- \| ---); 278 \| (--- \| --- \| ---); 279 \| (--- \| --- \| ---); 280 \| (--- \| --- \| ---); 281 \| (--- \| --- \| ---); 282 \| (--- \| --- \| ---); 283 \| (--- \| --- \| ---); 284 \| (--- \| --- \| ---); 285 \| (--- \| --- \| ---); 286 \| (--- \| --- \| ---); 287 \| (--- \| --- \| ---); 288 \| (--- \| --- \| ---); 289 \| (--- \| --- \| ---); 290 \| (--- \| --- \| ---); 291 \| (--- \| --- \| ---); 292 \| (--- \| --- \| ---); 293 \| (--- \| --- \| ---); 294 \| (--- \| --- \| ---); 295 \| (--- \| --- \| ---); 296 \| (--- \| --- \| ---); 297 \| (--- \| --- \| ---); 298 \| (--- \| --- \| ---); 299 \| (--- \| --- \| ---); 300 \| (--- \| --- \| ---); 301 \| (--- \| --- \| ---); 302 \| (--- \| --- \| ---); 303 \| (--- \| --- \| ---); 304 \| (--- \| --- \| ---); 305 \| (--- \| --- \| ---); 306 \| (--- \| --- \| ---); 307 \| (--- \| --- \| ---); 308 \| (--- \| --- \| ---); 309 \| (--- \| --- \| ---); 310 \| (--- \| --- \| ---); 311 \| (--- \| --- \| ---); 312 \| (--- \| --- \| ---); 313 \| (--- \| --- \| ---); 314 \| (--- \| --- \| ---); 315 \| (--- \| --- \| ---); 316 \| (--- \| --- \| ---); 317 \| (--- \| --- \| ---); 318 \| (--- \| --- \| ---); 319 \| (--- \| --- \| ---); 320 \| (--- \| --- \| ---); 321 \| (--- \| --- \| ---); 322 \| (--- \| --- \| ---); 323 \| (--- \| --- \| ---); 324 \| (--- \| --- \| ---); 325 \| (--- \| --- \| ---); 326 \| (--- \| --- \| ---); 327 \| (--- \| --- \| ---); 328 \| (--- \| --- \| ---); 329 \| (--- \| --- \| ---); 330 \| (--- \| --- \| ---); 331 \| (--- \| --- \| ---); 332 \| (--- \| --- \| ---); 333 \| (--- \| --- \| ---); 334 \| (--- \| --- \| ---); 335 \| (--- \| --- \| ---); 336 \| (--- \| --- \| ---); 337 \| (--- \| --- \| ---); 338 \| (--- \| --- \| ---); 339 \| (--- \| --- \| ---); 340 \| (--- \| --- \| ---); 341 \| (--- \| --- \| ---); 342 \| (--- \| --- \| ---); 343 \| (--- \| --- \| ---); 344 \| (--- \| --- \| ---); 345 \| (--- \| --- \| ---); 346 \| (--- \| --- \| ---); 347 \| (--- \| --- \| ---); 348 \| (--- \| --- \| ---); 349 \| (--- \| --- \| ---); 350 \| (--- \| --- \| ---); 351 \| (--- \| --- \| ---); 352 \| (--- \| --- \| ---); 353 \| (--- \| --- \| ---); 354 \| (--- \| --- \| ---); 355 \| (--- \| --- \| ---); 356 \| (--- \| --- \| ---); 357 \| (--- \| --- \| ---); 358 \| (--- \| --- \| ---); 359 \| (--- \| --- \| ---); 360 \| (--- \| --- \| ---); 361 \| (--- \| --- \| ---); 362 \| (--- \| --- \| ---); 363 \| (--- \| --- \| ---); 364 \| (--- \| --- \| ---); 365 \| (--- \| --- \| ---); 366 \| (--- \| --- \| ---); 367 \| (--- \| --- \| ---); 368 \| (--- \| --- \| ---); 369 \| (--- \| --- \| ---); 370 \| (--- \| --- \| ---); 371 \| (--- \| --- \| ---); 372 \| (--- \| --- \| ---); 373 \| (--- \| --- \| ---); 374 \| (--- \| --- \| ---); 375 \| (--- \| --- \| ---); 376 \| (--- \| --- \| ---); 377 \| (--- \| --- \| ---); 378 \| (--- \| --- \| ---); 379 \| (--- \| --- \| ---); 380 \| (--- \| --- \| ---); 381 \| (--- \| --- \| ---); 382 \| (--- \| --- \| ---); 383 \| (--- \| --- \| ---); 384 \| (--- \| --- \| ---); 385 \| (--- \| --- \| ---); 386 \| (--- \| --- \| ---); 387 \| (--- \| --- \| ---); 388 \| (--- \| --- \| ---); 389 \| (--- \| --- \| ---); 390 \| (--- \| --- \| ---); 391 \| (--- \| --- \| ---); 392 \| (--- \| --- \| ---); 393 \| (--- \| --- \| ---); 394 \| (--- \| --- \| ---); 395 \| (--- \| --- \| ---); 396 \| (--- \| --- \| ---); 397 \| (--- \| --- \| ---); 398 \| (--- \| --- \| ---); 399 \| (--- \| --- \| ---); 400 \| (--- \| --- \| ---); 401 \| (--- \| --- \| ---); 402 \| (--- \| --- \| ---); 403 \| (--- \| --- \| ---); 404 \| (--- \| --- \| ---); 405 \| (--- \| --- \| ---); 406 \| (--- \| --- \| ---); 407 \| (--- \| --- \| ---); 408 \| (--- \| --- \| ---); 409 \| (--- \| --- \| ---); 410 \| (--- \| --- \| ---); 411 \| (--- \| --- \| ---); 412 \| (--- \| --- \| ---); 413 \| (--- \| --- \| ---); 414 \| (--- \| --- \| ---); 415 \| (--- \| --- \| ---); 416 \| (--- \| --- \| ---); 417 \| (--- \| --- \| ---); 418 \| (--- \| --- \| ---); 419 \| (--- \| --- \| ---); 420 \| (--- \| --- \| ---); 421 \| (--- \| --- \| ---); 422 \| (--- \| --- \| ---); 423 \| (--- \| --- \| ---); 424 \| (--- \| --- \| ---); 425 \| (--- \| --- \| ---); 426 \| (--- \| --- \| ---); 427 \| (--- \| --- \| ---); 428 \| (--- \| --- \| ---); 429 \| (--- \| --- \| ---); 430 \| (--- \| --- \| ---); 431 \| (--- \| --- \| ---); 432 \| (--- \| --- \| ---); 433 \| (--- \| --- \| ---); 434 \| (--- \| --- \| ---); 435 \| (--- \| --- \| ---); 436 \| (--- \| --- \| ---); 437 \| (--- \| --- \| ---); 438 \| (--- \| --- \| ---); 439 \| (--- \| --- \| ---); 440 \| (--- \| --- \| ---); 441 \| (--- \| --- \| ---); 442 \| (--- \| --- \| ---); 443 \| (--- \| --- \| ---); 444 \| (--- \| --- \| ---); 445 \| (--- \| --- \| ---); 446 \| (--- \| --- \| ---); 447 \| (--- \| --- \| ---); 448 \| (--- \| --- \| ---); 449 \| (--- \| --- \| ---); 450 \| (--- \| --- \| ---); 451 \| (--- \| --- \| ---); 452 \| (--- \| --- \| ---); 453 \| (--- \| --- \| ---); 454 \| (--- \| --- \| ---); 455 \| (--- \| --- \| ---); 456 \| (--- \| --- \| ---); 457 \| (--- \| --- \| ---); 458 \| (--- \| --- \| ---); 459 \| (--- \| --- \| ---); 460 \| (--- \| --- \| ---); 461 \| (--- \| --- \| ---); 462 \| (--- \| --- \| ---); 463 \| (--- \| --- \| ---); 464 \| (--- \| --- \| ---); 465 \| (--- \| --- \| ---); 466 \| (--- \| --- \| ---); 467 \| (--- \| --- \| ---); 468 \| (--- \| --- \| ---); 469 \| (--- \| --- \| ---); 470 \| (--- \| --- \| ---); 471 \| (--- \| --- \| ---); 472 \| (--- \| --- \| ---); 473 \| (--- \| --- \| ---); 474 \| (--- \| --- \| ---); 475 \| (--- \| --- \| ---); 476 \| (--- \| --- \| ---); 477 \| (--- \| --- \| ---); 478 \| (--- \| --- \| ---); 479 \| (--- \| --- \| ---); 480 \| (--- \| --- \| ---); 481 \| (--- \| --- \| ---); 482 \| (--- \| --- \| ---); 483 \| (--- \| --- \| ---); 484 \| (--- \| --- \| ---); 485 \| (--- \| --- \| ---); 486 \| (--- \| --- \| ---); 487 \| (--- \| --- \| ---); 488 \| (--- \| --- \| ---); 489 \| (--- \| --- \| ---); 490 \| (--- \| --- \| ---); 491 \| (--- \| --- \| ---); 492 \| (--- \| --- \| ---); 493 \| (--- \| --- \| ---); 494 \| (--- \| --- \| ---); 495 \| (--- \| --- \| ---); 496 \| (--- \| --- \| ---); 497 \| (--- \| --- \| ---); 498 \| (--- \| --- \| ---); 499 \| (--- \| --- \| ---)) |

|  |  |
| --- | --- |
| Detailed template information [Hide] |  |

|  |  |  |  |
| --- | --- | --- | --- |
|  |  |  |  |

| # | Template | Alignment Coverage | 3D Model | Confidence | % i.d. | Template Information |
| --- | --- | --- | --- | --- | --- | --- |

|  |  |  |  |  |  |  |
| --- | --- | --- | --- | --- | --- | --- |
| 1 | c6g75B\_ | --- \| --- \| --- |  | 100.0 | 24 |


 **PDB header:**hydrolase **Chain:** B: **PDB Molecule:**common ancestor of haloalkane dehalogenase and renilla **PDBTitle:** crystal structure of the common ancestor of haloalkane dehalogenases2 and renilla luciferase (anchld-rluc) **PDB Entry:** PDBe RCSB PDBj |
| 2 | c2vf2A\_ | --- \| --- \| --- |  | 100.0 | 24 |


 **PDB header:**hydrolase **Chain:** A: **PDB Molecule:**2-hydroxy-6-oxo-6-phenylhexa-2,4-dienoate **PDBTitle:** x-ray crystal structure of hsad from mycobacterium2 tuberculosis **PDB Entry:** PDBe RCSB PDBj |
| 3 | c4mj3B\_ | --- \| --- \| --- |  | 100.0 | 22 |


 (**PDB header:**hydrolase **Chain:** B: **PDB Molecule:**haloalkane dehalogenase; **PDBTitle:** haloalkane dehalogenase dmra from mycobacterium rhodesiae js60 **PDB Entry:** PDBe RCSB PDBj) |
| 4 | d1bn7a\_ | --- \| --- \| --- |  | 100.0 | 23 | **Fold:** alpha/beta-Hydrolases **Superfamily:** alpha/beta-Hydrolases **Family:** Haloalkane dehalogenase **PDB entry:** PDBe RCSB PDBj |
| 5 | c3i28A\_ | --- \| --- \| --- |  | 100.0 | 20 |


 (**PDB header:**hydrolase **Chain:** A: **PDB Molecule:**epoxide hydrolase 2; **PDBTitle:** crystal structure of soluble epoxide hydrolase **PDB Entry:** PDBe RCSB PDBj) |
| 6 | c1cr6A\_ | --- \| --- \| --- |  | 100.0 | 20 |


 (**PDB header:**hydrolase **Chain:** A: **PDB Molecule:**epoxide hydrolase; **PDBTitle:** crystal structure of murine soluble epoxide hydrolase2 complexed with cpu inhibitor **PDB Entry:** PDBe RCSB PDBj) |
| 7 | c6y9gB\_ | --- \| --- \| --- |  | 100.0 | 22 |


 (**PDB header:**hydrolase **Chain:** B: **PDB Molecule:**ancestral haloalkane dehalogenase anchld5; **PDBTitle:** crystal structure of putative ancestral haloalkane dehalogenase2 anchld5 (node 5) **PDB Entry:** PDBe RCSB PDBj) |
| 8 | c4ns4A\_ | --- \| --- \| --- |  | 100.0 | 21 |


 (**PDB header:**hydrolase **Chain:** A: **PDB Molecule:**alpha/beta hydrolase fold protein; **PDBTitle:** crystal structure of cold-active estarase from psychrobacter2 cryohalolentis k5t **PDB Entry:** PDBe RCSB PDBj) |
| 9 | d1mj5a\_ | --- \| --- \| --- |  | 100.0 | 20 | **Fold:** alpha/beta-Hydrolases **Superfamily:** alpha/beta-Hydrolases **Family:** Haloalkane dehalogenase **PDB entry:** PDBe RCSB PDBj |
| 10 | c3u1tA\_ | --- \| --- \| --- |  | 100.0 | 21 |


 (**PDB header:**hydrolase **Chain:** A: **PDB Molecule:**dmma haloalkane dehalogenase; **PDBTitle:** haloalkane dehalogenase, dmma, of marine microbial origin **PDB Entry:** PDBe RCSB PDBj) |
| 11 | c4c6hA\_ | --- \| --- \| --- |  | 100.0 | 21 |


 (**PDB header:**hydrolase **Chain:** A: **PDB Molecule:**haloalkane dehalogenase; **PDBTitle:** haloalkane dehalogenase with 1-hexanol **PDB Entry:** PDBe RCSB PDBj) |
| 12 | c3a2nF\_ | --- \| --- \| --- |  | 100.0 | 20 |


 (**PDB header:**hydrolase **Chain:** F: **PDB Molecule:**haloalkane dehalogenase; **PDBTitle:** crystal structure of dbja (wild type type ii p21) **PDB Entry:** PDBe RCSB PDBj) |
| 13 | c3wibB\_ | --- \| --- \| --- |  | 100.0 | 20 |


 (**PDB header:**hydrolase **Chain:** B: **PDB Molecule:**haloalkane dehalogenase; **PDBTitle:** crystal structure of y109w mutant haloalkane dehalogenase data from2 agrobacterium tumefaciens c58 **PDB Entry:** PDBe RCSB PDBj) |
| 14 | c7jqzE\_ | --- \| --- \| --- |  | 100.0 | 21 |


 (**PDB header:**hydrolase **Chain:** E: **PDB Molecule:**alpha/beta hydrolase fold; **PDBTitle:** crystal structure of cfl2 wild-type from burkholderia cenocepacia **PDB Entry:** PDBe RCSB PDBj) |
| 15 | d1cr6a2 | --- \| --- \| --- |  | 100.0 | 20 | **Fold:** alpha/beta-Hydrolases **Superfamily:** alpha/beta-Hydrolases **Family:** Epoxide hydrolase **PDB entry:** PDBe RCSB PDBj |
| 16 | d1zd3a2 | --- \| --- \| --- |  | 100.0 | 20 | **Fold:** alpha/beta-Hydrolases **Superfamily:** alpha/beta-Hydrolases **Family:** Epoxide hydrolase **PDB entry:** PDBe RCSB PDBj |
| 17 | c2e3jA\_ | --- \| --- \| --- |  | 100.0 | 20 |


 (**PDB header:**hydrolase **Chain:** A: **PDB Molecule:**epoxide hydrolase ephb; **PDBTitle:** the crystal structure of epoxide hydrolase b (rv1938) from2 mycobacterium tuberculosis at 2.1 angstrom **PDB Entry:** PDBe RCSB PDBj) |
| 18 | c7avrG\_ | --- \| --- \| --- |  | 100.0 | 18 |


 (**PDB header:**hydrolase **Chain:** G: **PDB Molecule:**haloalkane dehalogenase 1; **PDBTitle:** the tetrameric structure of haloalkane dehalogenase dpaa from2 paraglaciecola agarilytica no2 **PDB Entry:** PDBe RCSB PDBj) |
| 19 | c2qvbA\_ | --- \| --- \| --- |  | 100.0 | 20 |


 (**PDB header:**hydrolase **Chain:** A: **PDB Molecule:**haloalkane dehalogenase 3; **PDBTitle:** crystal structure of haloalkane dehalogenase rv2579 from mycobacterium2 tuberculosis **PDB Entry:** PDBe RCSB PDBj) |
| 20 | c5xmdA\_ | --- \| --- \| --- |  | 100.0 | 19 |


 (**PDB header:**hydrolase **Chain:** A: **PDB Molecule:**epoxide hydrolase a; **PDBTitle:** crystal structure of epoxide hydrolase vreh1 from vigna radiata **PDB Entry:** PDBe RCSB PDBj) |
| 21 | d1hkha\_ | --- \| --- \| --- | not modelled | 100.0 | 20 | **Fold:** alpha/beta-Hydrolases **Superfamily:** alpha/beta-Hydrolases **Family:** Haloperoxidase **PDB entry:** PDBe RCSB PDBj |
| 22 | c2pseA\_ | --- \| --- \| --- | not modelled | 100.0 | 18 |


 (**PDB header:**oxidoreductase **Chain:** A: **PDB Molecule:**renilla-luciferin 2-monooxygenase; **PDBTitle:** crystal structures of the luciferase and green fluorescent protein2 from renilla reniformis **PDB Entry:** PDBe RCSB PDBj) |
| 23 | c5ng7B\_ | --- \| --- \| --- | not modelled | 100.0 | 20 |


 (**PDB header:**hydrolase **Chain:** B: **PDB Molecule:**epoxide hydrolase; **PDBTitle:** novel epoxide hydrolases belonging to the alpha/beta hydrolases2 superfamily in metagenomes from hot environments **PDB Entry:** PDBe RCSB PDBj) |
| 24 | d2rhwa1 | --- \| --- \| --- | not modelled | 100.0 | 19 | **Fold:** alpha/beta-Hydrolases **Superfamily:** alpha/beta-Hydrolases **Family:** Carbon-carbon bond hydrolase **PDB entry:** PDBe RCSB PDBj |
| 25 | c4psuA\_ | --- \| --- \| --- | not modelled | 100.0 | 19 |


 (**PDB header:**hydrolase **Chain:** A: **PDB Molecule:**alpha/beta hydrolase; **PDBTitle:** crystal structure of alpha/beta hydrolase from rhodopseudomonas2 palustris cga009 **PDB Entry:** PDBe RCSB PDBj) |
| 26 | d1a8qa\_ | --- \| --- \| --- | not modelled | 100.0 | 20 | **Fold:** alpha/beta-Hydrolases **Superfamily:** alpha/beta-Hydrolases **Family:** Haloperoxidase **PDB entry:** PDBe RCSB PDBj |
| 27 | c6f9oA\_ | --- \| --- \| --- | not modelled | 100.0 | 18 |


 (**PDB header:**hydrolase **Chain:** A: **PDB Molecule:**haloalkane dehalogenase; **PDBTitle:** crystal structure of cold-adapted haloalkane dehalogenase dpca from2 psychrobacter cryohalolentis k5 **PDB Entry:** PDBe RCSB PDBj) |
| 28 | c2wirB\_ | --- \| --- \| --- | not modelled | 98.8 | 20 |


 (**PDB header:**hydrolase **Chain:** B: **PDB Molecule:**alpha/beta hydrolase fold-3 domain protein; **PDBTitle:** hyperthermophilic esterase from the archeon pyrobaculum2 calidifontis **PDB Entry:** PDBe RCSB PDBj) |
| 29 | c7wwfD\_ | --- \| --- \| --- | not modelled | 100.0 | 19 |


 (**PDB header:**biosynthetic protein **Chain:** D: **PDB Molecule:**esterase; **PDBTitle:** crystal structure of bioh3 from mycolicibacterium smegmatis **PDB Entry:** PDBe RCSB PDBj) |
| 30 | c5frdA\_ | --- \| --- \| --- | not modelled | 100.0 | 20 |


 (**PDB header:**hydrolase **Chain:** A: **PDB Molecule:**carboxylesterase (est-2); **PDBTitle:** structure of a thermophilic esterase **PDB Entry:** PDBe RCSB PDBj) |
| 31 | c6i8wB\_ | --- \| --- \| --- | not modelled | 100.0 | 16 |


 (**PDB header:**hydrolase **Chain:** B: **PDB Molecule:**alpha/beta fold hydrolase; **PDBTitle:** crystal structure of a membrane phospholipase a, a novel bacterial2 virulence factor **PDB Entry:** PDBe RCSB PDBj) |
| 32 | c4opmB\_ | --- \| --- \| --- | not modelled | 100.0 | 17 |


 (**PDB header:**hydrolase **Chain:** B: **PDB Molecule:**lipase; **PDBTitle:** crystal structure of a putative lipase (lip1) from acinetobacter2 baumannii aye at 1.70 a resolution **PDB Entry:** PDBe RCSB PDBj) |
| 33 | c4inzB\_ | --- \| --- \| --- | not modelled | 100.0 | 19 |


 (**PDB header:**hydrolase **Chain:** B: **PDB Molecule:**soluble epoxide hydrolase; **PDBTitle:** the crystal structure of m145a mutant of an epoxide hydrolase from2 bacillus megaterium **PDB Entry:** PDBe RCSB PDBj) |
| 34 | c3qitB\_ | --- \| --- \| --- | not modelled | 100.0 | 19 |


 (**PDB header:**hydrolase **Chain:** B: **PDB Molecule:**polyketide synthase; **PDBTitle:** thioesterase domain from curacin biosynthetic pathway **PDB Entry:** PDBe RCSB PDBj) |
| 35 | c2xt0A\_ | --- \| --- \| --- | not modelled | 100.0 | 18 |


 (**PDB header:**hydrolase **Chain:** A: **PDB Molecule:**haloalkane dehalogenase; **PDBTitle:** dehalogenase dppa from plesiocystis pacifica sir-i **PDB Entry:** PDBe RCSB PDBj) |
| 36 | c5w8pA\_ | --- \| --- \| --- | not modelled | 100.0 | 16 |


 (**PDB header:**transferase **Chain:** A: **PDB Molecule:**homoserine o-acetyltransferase; **PDBTitle:** homoserine transacetylase metx from mycobacterium abscessus **PDB Entry:** PDBe RCSB PDBj) |
| 37 | c1u2eA\_ | --- \| --- \| --- | not modelled | 100.0 | 18 |


 **PDB header:**hydrolase **Chain:** A: **PDB Molecule:**2-hydroxy-6-ketonona-2,4-dienedioic acid **PDBTitle:** crystal structure of the c-c bond hydrolase mhpc **PDB Entry:** PDBe RCSB PDBj |
| 38 | c6kxhD\_ | --- \| --- \| --- | not modelled | 100.0 | 19 |


 (**PDB header:**hydrolase **Chain:** D: **PDB Molecule:**putative hydrolase; **PDBTitle:** alp1u\_y247f mutant in complex with fluostatin c **PDB Entry:** PDBe RCSB PDBj) |
| 39 | c6unwA\_ | --- \| --- \| --- | not modelled | 100.0 | 18 |


 (**PDB header:**hydrolase **Chain:** A: **PDB Molecule:**soluble epoxide hydrolase; **PDBTitle:** epoxide hydrolase from an endophytic streptomyces **PDB Entry:** PDBe RCSB PDBj) |
| 40 | c5xb6D\_ | --- \| --- \| --- | not modelled | 99.9 | 18 |


 (**PDB header:**hydrolase **Chain:** D: **PDB Molecule:**uncharacterized protein ycjy; **PDBTitle:** crystal structure of ycjy from e. coli **PDB Entry:** PDBe RCSB PDBj) |
| 41 | c5cw2C\_ | --- \| --- \| --- | not modelled | 100.0 | 18 |


 (**PDB header:**hydrolase **Chain:** C: **PDB Molecule:**putative epoxide hydrolase epha; **PDBTitle:** crystal structure of epoxide hydrolase a from mycobacterium2 thermoresistibile **PDB Entry:** PDBe RCSB PDBj) |
| 42 | d2hu7a2 | --- \| --- \| --- | not modelled | 99.6 | 19 | **Fold:** alpha/beta-Hydrolases **Superfamily:** alpha/beta-Hydrolases **Family:** Acylamino-acid-releasing enzyme, C-terminal donain **PDB entry:** PDBe RCSB PDBj |
| 43 | c4b9aA\_ | --- \| --- \| --- | not modelled | 100.0 | 17 |


 (**PDB header:**hydrolase **Chain:** A: **PDB Molecule:**probable epoxide hydrolase; **PDBTitle:** structure of a putative epoxide hydrolase from pseudomonas2 aeruginosa. **PDB Entry:** PDBe RCSB PDBj) |
| 44 | c8e18A\_ | --- \| --- \| --- | not modelled | 99.9 | 15 |


 (**PDB header:**hydrolase **Chain:** A: **PDB Molecule:**secreted hydrolase; **PDBTitle:** crystal structure of apo tnmk1 **PDB Entry:** PDBe RCSB PDBj) |
| 45 | c5jrlC\_ | --- \| --- \| --- | not modelled | 99.3 | 20 |


 **PDB header:**hydrolase **Chain:** C: **PDB Molecule:**dipeptidyl aminopeptidases/acylaminoacyl-peptidases-like **PDBTitle:** crystal structure of the sphingopyxin i lasso peptide isopeptidase2 spi-isop (native) **PDB Entry:** PDBe RCSB PDBj |
| 46 | c4zxiA\_ | --- \| --- \| --- | not modelled | 99.4 | 16 |


 (**PDB header:**biosynthetic protein **Chain:** A: **PDB Molecule:**tyrocidine synthetase 3; **PDBTitle:** crystal structure of holo-ab3403 a four domain nonribosomal peptide2 synthetase bound to amp and glycine **PDB Entry:** PDBe RCSB PDBj) |
| 47 | d1brta\_ | --- \| --- \| --- | not modelled | 100.0 | 18 | **Fold:** alpha/beta-Hydrolases **Superfamily:** alpha/beta-Hydrolases **Family:** Haloperoxidase **PDB entry:** PDBe RCSB PDBj |
| 48 | d1fj2a\_ | --- \| --- \| --- | not modelled | 99.8 | 22 | **Fold:** alpha/beta-Hydrolases **Superfamily:** alpha/beta-Hydrolases **Family:** Carboxylesterase/thioesterase 1 **PDB entry:** PDBe RCSB PDBj |
| 49 | c4uhhA\_ | --- \| --- \| --- | not modelled | 100.0 | 17 |


 (**PDB header:**hydrolase **Chain:** A: **PDB Molecule:**esterase; **PDBTitle:** structural studies of a thermophilic esterase from2 thermogutta terrifontis (cacodylate complex) **PDB Entry:** PDBe RCSB PDBj) |
| 50 | d1qo7a\_ | --- \| --- \| --- | not modelled | 100.0 | 18 | **Fold:** alpha/beta-Hydrolases **Superfamily:** alpha/beta-Hydrolases **Family:** Epoxide hydrolase **PDB entry:** PDBe RCSB PDBj |
| 51 | c3kdaB\_ | --- \| --- \| --- | not modelled | 100.0 | 18 |


 (**PDB header:**hydrolase **Chain:** B: **PDB Molecule:**cftr inhibitory factor (cif); **PDBTitle:** crystal structure of the cftr inhibitory factor cif with the h269a2 mutation **PDB Entry:** PDBe RCSB PDBj) |
| 52 | c4rncB\_ | --- \| --- \| --- | not modelled | 100.0 | 18 |


 (**PDB header:**hydrolase **Chain:** B: **PDB Molecule:**esterase; **PDBTitle:** crystal structure of an esterase rhest1 from rhodococcus sp. ecu1013 **PDB Entry:** PDBe RCSB PDBj) |
| 53 | c6u2mC\_ | --- \| --- \| --- | not modelled | 100.0 | 22 |


 (**PDB header:**hydrolase **Chain:** C: **PDB Molecule:**halocamp v2; **PDBTitle:** crystal structure of a halotag-based calcium indicator, halocamp v2,2 bound to jf635 **PDB Entry:** PDBe RCSB PDBj) |
| 54 | c4d9jI\_ | --- \| --- \| --- | not modelled | 100.0 | 16 |


 **PDB header:**de novo protein **Chain:** I: **PDB Molecule:**designed 16nm tetrahedral protein cage containing non-haem **PDBTitle:** structure of a 16 nm protein cage designed by fusing symmetric2 oligomeric domains **PDB Entry:** PDBe RCSB PDBj |
| 55 | c5uohA\_ | --- \| --- \| --- | not modelled | 99.9 | 15 |


 (**PDB header:**hydrolase **Chain:** A: **PDB Molecule:**carboxylesterase a; **PDBTitle:** crystal structure of hip1 (rv2224c) t466a mutant **PDB Entry:** PDBe RCSB PDBj) |
| 56 | d1f0na\_ | --- \| --- \| --- | not modelled | 99.5 | 18 | **Fold:** alpha/beta-Hydrolases **Superfamily:** alpha/beta-Hydrolases **Family:** Mycobacterial antigens **PDB entry:** PDBe RCSB PDBj |
| 57 | c6aaeA\_ | --- \| --- \| --- | not modelled | 98.9 | 19 |


 (**PDB header:**hydrolase **Chain:** A: **PDB Molecule:**esterase; **PDBTitle:** crystal structure of chloramphenicol-metabolizaing enzyme estdl136 **PDB Entry:** PDBe RCSB PDBj) |
| 58 | c5hk8A\_ | --- \| --- \| --- | not modelled | 100.0 | 19 |


 (**PDB header:**hydrolase **Chain:** A: **PDB Molecule:**probable pheophorbidase; **PDBTitle:** crystal structure of a methylesterase protein mes16 from arabidopsis **PDB Entry:** PDBe RCSB PDBj) |
| 59 | c6ojdB\_ | --- \| --- \| --- | not modelled | 100.0 | 20 |


 (**PDB header:**hydrolase **Chain:** B: **PDB Molecule:**nocb; **PDBTitle:** a high-resolution crystal structure of covalent complex of nocb2 thioesterase domain with fluorophosphonate nocardicin g analog **PDB Entry:** PDBe RCSB PDBj) |
| 60 | c4lxgA\_ | --- \| --- \| --- | not modelled | 100.0 | 17 |


 (**PDB header:**hydrolase **Chain:** A: **PDB Molecule:**mcp hydrolase; **PDBTitle:** crystal structure of dxnb2, a carbon - carbon bond hydrolase from2 sphingomonas wittichii rw1 **PDB Entry:** PDBe RCSB PDBj) |
| 61 | c5yhpB\_ | --- \| --- \| --- | not modelled | 100.0 | 17 |


 (**PDB header:**hydrolase **Chain:** B: **PDB Molecule:**cold active proline iminopeptidase; **PDBTitle:** proline iminopeptidase from psychrophilic yeast glaciozyma antarctica **PDB Entry:** PDBe RCSB PDBj) |
| 62 | c3u0vA\_ | --- \| --- \| --- | not modelled | 99.6 | 21 |


 (**PDB header:**hydrolase **Chain:** A: **PDB Molecule:**lysophospholipase-like protein 1; **PDBTitle:** crystal structure analysis of human lyplal1 **PDB Entry:** PDBe RCSB PDBj) |
| 63 | d1b6ga\_ | --- \| --- \| --- | not modelled | 100.0 | 15 | **Fold:** alpha/beta-Hydrolases **Superfamily:** alpha/beta-Hydrolases **Family:** Haloalkane dehalogenase **PDB entry:** PDBe RCSB PDBj |
| 64 | c1y37A\_ | --- \| --- \| --- | not modelled | 100.0 | 16 |


 (**PDB header:**hydrolase **Chain:** A: **PDB Molecule:**fluoroacetate dehalogenase; **PDBTitle:** structure of fluoroacetate dehalogenase from burkholderia sp. fa1 **PDB Entry:** PDBe RCSB PDBj) |
| 65 | c7dq9A\_ | --- \| --- \| --- | not modelled | 100.0 | 17 |


 **PDB header:**hydrolase **Chain:** A: **PDB Molecule:**predicted hydrolases or acyltransferases (alpha/beta **PDBTitle:** crystal structure of a type-a feruloyl esterase from gut alistipes2 shahii **PDB Entry:** PDBe RCSB PDBj |
| 66 | d1ivya\_ | --- \| --- \| --- | not modelled | 99.0 | 17 | **Fold:** alpha/beta-Hydrolases **Superfamily:** alpha/beta-Hydrolases **Family:** Serine carboxypeptidase-like **PDB entry:** PDBe RCSB PDBj |
| 67 | c2zyiB\_ | --- \| --- \| --- | not modelled | 99.8 | 19 |


 (**PDB header:**hydrolase **Chain:** B: **PDB Molecule:**lipase, putative; **PDBTitle:** a. fulgidus lipase with fatty acid fragment and calcium **PDB Entry:** PDBe RCSB PDBj) |
| 68 | c5mxpB\_ | --- \| --- \| --- | not modelled | 100.0 | 17 |


 (**PDB header:**hydrolase **Chain:** B: **PDB Molecule:**alpha/beta hydrolase; **PDBTitle:** haloalkane dehalogenase dmxa from marinobacter sp. elb17 possessing a2 unique catalytic residue **PDB Entry:** PDBe RCSB PDBj) |
| 69 | c3r3xA\_ | --- \| --- \| --- | not modelled | 100.0 | 16 |


 (**PDB header:**hydrolase **Chain:** A: **PDB Molecule:**fluoroacetate dehalogenase; **PDBTitle:** crystal structure of the fluoroacetate dehalogenase rpa1163 -2 asp110asn/bromoacetate **PDB Entry:** PDBe RCSB PDBj) |
| 70 | c2hdwB\_ | --- \| --- \| --- | not modelled | 99.9 | 16 |


 (**PDB header:**hydrolase **Chain:** B: **PDB Molecule:**hypothetical protein pa2218; **PDBTitle:** crystal structure of hypothetical protein pa2218 from pseudomonas2 aeruginosa **PDB Entry:** PDBe RCSB PDBj) |
| 71 | d1wpxa1 | --- \| --- \| --- | not modelled | 98.8 | 15 | **Fold:** alpha/beta-Hydrolases **Superfamily:** alpha/beta-Hydrolases **Family:** Serine carboxypeptidase-like **PDB entry:** PDBe RCSB PDBj |
| 72 | c7c4dA\_ | --- \| --- \| --- | not modelled | 100.0 | 17 |


 (**PDB header:**hydrolase **Chain:** A: **PDB Molecule:**putative esterase; **PDBTitle:** marine microorganism esterase **PDB Entry:** PDBe RCSB PDBj) |
| 73 | c2y6vB\_ | --- \| --- \| --- | not modelled | 100.0 | 15 |


 (**PDB header:**hydrolase **Chain:** B: **PDB Molecule:**peroxisomal membrane protein lpx1; **PDBTitle:** peroxisomal alpha-beta-hydrolase lpx1 (yor084w) from saccharomyces2 cerevisiae (crystal form i) **PDB Entry:** PDBe RCSB PDBj) |
| 74 | c3i1iA\_ | --- \| --- \| --- | not modelled | 100.0 | 14 |


 (**PDB header:**transferase **Chain:** A: **PDB Molecule:**homoserine o-acetyltransferase; **PDBTitle:** x-ray crystal structure of homoserine o-acetyltransferase from2 bacillus anthracis **PDB Entry:** PDBe RCSB PDBj) |
| 75 | d1uk8a\_ | --- \| --- \| --- | not modelled | 100.0 | 16 | **Fold:** alpha/beta-Hydrolases **Superfamily:** alpha/beta-Hydrolases **Family:** Carbon-carbon bond hydrolase **PDB entry:** PDBe RCSB PDBj |
| 76 | d1e89a\_ | --- \| --- \| --- | not modelled | 100.0 | 17 | **Fold:** alpha/beta-Hydrolases **Superfamily:** alpha/beta-Hydrolases **Family:** Hydroxynitrile lyase-like **PDB entry:** PDBe RCSB PDBj |
| 77 | c5nfqA\_ | --- \| --- \| --- | not modelled | 100.0 | 16 |


 **PDB header:**hydrolase **Chain:** A: **PDB Molecule:**epoxide hydrolase belonging to alpha/beta hydrolase **PDBTitle:** novel epoxide hydrolases belonging to the alpha/beta hydrolases2 superfamily in metagenomes from hot environments **PDB Entry:** PDBe RCSB PDBj |
| 78 | c3kxpD\_ | --- \| --- \| --- | not modelled | 100.0 | 16 |


 (**PDB header:**hydrolase **Chain:** D: **PDB Molecule:**alpha-(n-acetylaminomethylene)succinic acid hydrolase; **PDBTitle:** crystal structure of e-2-(acetamidomethylene)succinate hydrolase **PDB Entry:** PDBe RCSB PDBj) |
| 79 | c6khlB\_ | --- \| --- \| --- | not modelled | 100.0 | 16 |


 (**PDB header:**hydrolase **Chain:** B: **PDB Molecule:**hydrolase, alpha/beta domain protein; **PDBTitle:** lipase (blocked form) **PDB Entry:** PDBe RCSB PDBj) |
| 80 | c1mo2A\_ | --- \| --- \| --- | not modelled | 100.0 | 20 |


 (**PDB header:**transferase **Chain:** A: **PDB Molecule:**erythronolide synthase, modules 5 and 6; **PDBTitle:** thioesterase domain from 6-deoxyerythronolide synthase (debs te), ph2 8.5 **PDB Entry:** PDBe RCSB PDBj) |
| 81 | d1mo2a\_ | --- \| --- \| --- | not modelled | 100.0 | 20 | **Fold:** alpha/beta-Hydrolases **Superfamily:** alpha/beta-Hydrolases **Family:** Thioesterase domain of polypeptide, polyketide and fatty acid synthases **PDB entry:** PDBe RCSB PDBj |
| 82 | c2o2gA\_ | --- \| --- \| --- | not modelled | 99.8 | 21 |


 (**PDB header:**hydrolase **Chain:** A: **PDB Molecule:**dienelactone hydrolase; **PDBTitle:** crystal structure of dienelactone hydrolase (yp\_324580.1) from2 anabaena variabilis atcc 29413 at 1.92 a resolution **PDB Entry:** PDBe RCSB PDBj) |
| 83 | c7wwhB\_ | --- \| --- \| --- | not modelled | 99.9 | 18 |


 (**PDB header:**hydrolase **Chain:** B: **PDB Molecule:**alpha/beta hydrolase; **PDBTitle:** crystal structure of the geobacillus thermoglucosidasius feruloyl2 esterase gthfae **PDB Entry:** PDBe RCSB PDBj) |
| 84 | c3wwoA\_ | --- \| --- \| --- | not modelled | 100.0 | 17 |


 (**PDB header:**lyase **Chain:** A: **PDB Molecule:**(s)-hydroxynitrile lyase; **PDBTitle:** s-selective hydroxynitrile lyase from baliospermum montanum (apo1) **PDB Entry:** PDBe RCSB PDBj) |
| 85 | c2hu7A\_ | --- \| --- \| --- | not modelled | 99.9 | 17 |


 (**PDB header:**hydrolase **Chain:** A: **PDB Molecule:**acylamino-acid-releasing enzyme; **PDBTitle:** binding of inhibitors by acylaminoacyl peptidase **PDB Entry:** PDBe RCSB PDBj) |
| 86 | d1q0ra\_ | --- \| --- \| --- | not modelled | 100.0 | 16 | **Fold:** alpha/beta-Hydrolases **Superfamily:** alpha/beta-Hydrolases **Family:** Aclacinomycin methylesterase RdmC **PDB entry:** PDBe RCSB PDBj |
| 87 | d1hlga\_ | --- \| --- \| --- | not modelled | 100.0 | 14 | **Fold:** alpha/beta-Hydrolases **Superfamily:** alpha/beta-Hydrolases **Family:** Gastric lipase **PDB entry:** PDBe RCSB PDBj |
| 88 | c7qjmA\_ | --- \| --- \| --- | not modelled | 100.0 | 15 |


 (**PDB header:**hydrolase **Chain:** A: **PDB Molecule:**alpha/beta-hydrolase (202); **PDBTitle:** crystal structure of an alpha/beta-hydrolase enzyme from chloroflexus2 sp. ms-g (202) **PDB Entry:** PDBe RCSB PDBj) |
| 89 | c5esrA\_ | --- \| --- \| --- | not modelled | 100.0 | 15 |


 (**PDB header:**hydrolase **Chain:** A: **PDB Molecule:**haloalkane dehalogenase; **PDBTitle:** crystal structure of haloalkane dehalogenase (dcca) from caulobacter2 crescentus **PDB Entry:** PDBe RCSB PDBj) |
| 90 | d1j1ia\_ | --- \| --- \| --- | not modelled | 100.0 | 17 | **Fold:** alpha/beta-Hydrolases **Superfamily:** alpha/beta-Hydrolases **Family:** Carbon-carbon bond hydrolase **PDB entry:** PDBe RCSB PDBj |
| 91 | c1j1iA\_ | --- \| --- \| --- | not modelled | 100.0 | 17 |


 (**PDB header:**hydrolase **Chain:** A: **PDB Molecule:**meta cleavage compound hydrolase; **PDBTitle:** crystal structure of a his-tagged serine hydrolase involved2 in the carbazole degradation (carc enzyme) **PDB Entry:** PDBe RCSB PDBj) |
| 92 | c2vsqA\_ | --- \| --- \| --- | not modelled | 99.0 | 14 |


 (**PDB header:**ligase **Chain:** A: **PDB Molecule:**surfactin synthetase subunit 3; **PDBTitle:** structure of surfactin a synthetase c (srfa-c), a nonribosomal peptide2 synthetase termination module **PDB Entry:** PDBe RCSB PDBj) |
| 93 | c6ix4A\_ | --- \| --- \| --- | not modelled | 100.0 | 16 |


 (**PDB header:**hydrolase **Chain:** A: **PDB Molecule:**microsomal epoxide hydrolase; **PDBTitle:** structure of an epoxide hydrolase from aspergillus usamii e001 (aueh2)2 at 1.51 angstroms resolution **PDB Entry:** PDBe RCSB PDBj) |
| 94 | c2qmqA\_ | --- \| --- \| --- | not modelled | 100.0 | 16 |


 (**PDB header:**signaling protein **Chain:** A: **PDB Molecule:**protein ndrg2; **PDBTitle:** crystal structure of a n-myc downstream regulated 2 protein (ndrg2,2 syld, ndr2, ai182517, au040374) from mus musculus at 1.70 a3 resolution **PDB Entry:** PDBe RCSB PDBj) |
| 95 | d1a88a\_ | --- \| --- \| --- | not modelled | 100.0 | 16 | **Fold:** alpha/beta-Hydrolases **Superfamily:** alpha/beta-Hydrolases **Family:** Haloperoxidase **PDB entry:** PDBe RCSB PDBj |
| 96 | d2h7xa1 | --- \| --- \| --- | not modelled | 100.0 | 19 | **Fold:** alpha/beta-Hydrolases **Superfamily:** alpha/beta-Hydrolases **Family:** Thioesterase domain of polypeptide, polyketide and fatty acid synthases **PDB entry:** PDBe RCSB PDBj |
| 97 | c4qlaA\_ | --- \| --- \| --- | not modelled | 100.0 | 16 |


 (**PDB header:**hydrolase **Chain:** A: **PDB Molecule:**juvenile hormone epoxide hydrolase; **PDBTitle:** crystal structure of juvenile hormone epoxide hydrolase from the2 silkworm bombyx mori **PDB Entry:** PDBe RCSB PDBj) |
| 98 | c6ra2E\_ | --- \| --- \| --- | not modelled | 100.0 | 16 |


 **PDB header:**hydrolase **Chain:** E: **PDB Molecule:**putative dioxygenase (1h-3-hydroxy-4-oxoquinaldine 2,4- **PDBTitle:** structural basis for recognition and ring-cleavage of the pseudomonas2 quinolone signal (pqs) by aqdc **PDB Entry:** PDBe RCSB PDBj |
| 99 | c7jqxD\_ | --- \| --- \| --- | not modelled | 100.0 | 15 |


 (**PDB header:**hydrolase **Chain:** D: **PDB Molecule:**cif-like 1 wild-type; **PDBTitle:** crystal structure of cfl1 wild-type from burkholderia cenocepacia **PDB Entry:** PDBe RCSB PDBj) |
| 100 | c4i3fA\_ | --- \| --- \| --- | not modelled | 100.0 | 15 |


 (**PDB header:**hydrolase **Chain:** A: **PDB Molecule:**serine hydrolase ccsp0084; **PDBTitle:** crystal structure of serine hydrolase ccsp0084 from the polyaromatic2 hydrocarbon (pah)-degrading bacterium cycloclasticus zankles **PDB Entry:** PDBe RCSB PDBj) |
| 101 | c6cl4A\_ | --- \| --- \| --- | not modelled | 99.9 | 18 |


 (**PDB header:**hydrolase **Chain:** A: **PDB Molecule:**lipase c12; **PDBTitle:** lipc12 - lipase from metagenomics **PDB Entry:** PDBe RCSB PDBj) |
| 102 | c3fobA\_ | --- \| --- \| --- | not modelled | 100.0 | 16 |


 (**PDB header:**oxidoreductase **Chain:** A: **PDB Molecule:**bromoperoxidase; **PDBTitle:** crystal structure of bromoperoxidase from bacillus anthracis **PDB Entry:** PDBe RCSB PDBj) |
| 103 | c5hdfB\_ | --- \| --- \| --- | not modelled | 100.0 | 16 |


 (**PDB header:**hydrolase **Chain:** B: **PDB Molecule:**hydrolase; **PDBTitle:** hydrolase semet-stna **PDB Entry:** PDBe RCSB PDBj) |
| 104 | d2pl5a1 | --- \| --- \| --- | not modelled | 100.0 | 14 | **Fold:** alpha/beta-Hydrolases **Superfamily:** alpha/beta-Hydrolases **Family:** O-acetyltransferase **PDB entry:** PDBe RCSB PDBj |
| 105 | c5efzC\_ | --- \| --- \| --- | not modelled | 100.0 | 14 |


 (**PDB header:**hydrolase **Chain:** C: **PDB Molecule:**homoserine o-acetyltransferase; **PDBTitle:** monoclinic structure of the acetyl esterase mekb **PDB Entry:** PDBe RCSB PDBj) |
| 106 | c4qlaB\_ | --- \| --- \| --- | not modelled | 100.0 | 16 |


 (**PDB header:**hydrolase **Chain:** B: **PDB Molecule:**juvenile hormone epoxide hydrolase; **PDBTitle:** crystal structure of juvenile hormone epoxide hydrolase from the2 silkworm bombyx mori **PDB Entry:** PDBe RCSB PDBj) |
| 107 | c6qe2A\_ | --- \| --- \| --- | not modelled | 100.0 | 16 |


 (**PDB header:**hydrolase **Chain:** A: **PDB Molecule:**monoacylglycerol lipase; **PDBTitle:** crystal structure of paleococcus ferrophilus monoacylglycerol lipase. **PDB Entry:** PDBe RCSB PDBj) |
| 108 | c4nvrC\_ | --- \| --- \| --- | not modelled | 100.0 | 15 |


 (**PDB header:**transferase **Chain:** C: **PDB Molecule:**putative acyltransferase; **PDBTitle:** 2.22 angstrom resolution crystal structure of a putative2 acyltransferase from salmonella enterica **PDB Entry:** PDBe RCSB PDBj) |
| 109 | c3hjuB\_ | --- \| --- \| --- | not modelled | 100.0 | 15 |


 (**PDB header:**hydrolase **Chain:** B: **PDB Molecule:**monoglyceride lipase; **PDBTitle:** crystal structure of human monoglyceride lipase **PDB Entry:** PDBe RCSB PDBj) |
| 110 | c3wzlB\_ | --- \| --- \| --- | not modelled | 100.0 | 16 |


 (**PDB header:**hydrolase **Chain:** B: **PDB Molecule:**zearalenone hydrolase; **PDBTitle:** zen lactonase **PDB Entry:** PDBe RCSB PDBj) |
| 111 | c6azdA\_ | --- \| --- \| --- | not modelled | 100.0 | 16 |


 (**PDB header:**hydrolase **Chain:** A: **PDB Molecule:**ppkai2-like h; **PDBTitle:** crystal structure of physcomitrella patens kai2-like h **PDB Entry:** PDBe RCSB PDBj) |
| 112 | d1va4a\_ | --- \| --- \| --- | not modelled | 100.0 | 16 | **Fold:** alpha/beta-Hydrolases **Superfamily:** alpha/beta-Hydrolases **Family:** Haloperoxidase **PDB entry:** PDBe RCSB PDBj |
| 113 | c7e04A\_ | --- \| --- \| --- | not modelled | 100.0 | 17 |


 (**PDB header:**hydrolase **Chain:** A: **PDB Molecule:**lipase; **PDBTitle:** crystal structure of bomgl, a monoacylglycerol lipase from marine2 bacillus sp. **PDB Entry:** PDBe RCSB PDBj) |
| 114 | c4q3kB\_ | --- \| --- \| --- | not modelled | 99.2 | 19 |


 (**PDB header:**hydrolase **Chain:** B: **PDB Molecule:**mgs-m1; **PDBTitle:** crystal structure of mgs-m1, an alpha/beta hydrolase enzyme from a2 medee basin deep-sea metagenome library **PDB Entry:** PDBe RCSB PDBj) |
| 115 | c5oluA\_ | --- \| --- \| --- | not modelled | 100.0 | 15 |


 (**PDB header:**hydrolase **Chain:** A: **PDB Molecule:**alpha/beta hydrolase family protein; **PDBTitle:** the crystal structure of a highly thermostable carboxyl esterase from2 bacillus coagulans in complex with glycerol **PDB Entry:** PDBe RCSB PDBj) |
| 116 | c5jd6A\_ | --- \| --- \| --- | not modelled | 100.0 | 19 |


 (**PDB header:**hydrolase **Chain:** A: **PDB Molecule:**mgs-mche2; **PDBTitle:** crystal structure of mgs-mche2, an alpha/beta hydrolase enzyme from2 the metagenome of sediments from the lagoon of mar chica, morocco **PDB Entry:** PDBe RCSB PDBj) |
| 117 | c4f0jA\_ | --- \| --- \| --- | not modelled | 100.0 | 14 |


 (**PDB header:**hydrolase **Chain:** A: **PDB Molecule:**probable hydrolytic enzyme; **PDBTitle:** crystal structure of a probable hydrolytic enzyme (pa3053) from2 pseudomonas aeruginosa pao1 at 1.50 a resolution **PDB Entry:** PDBe RCSB PDBj) |
| 118 | d1imja\_ | --- \| --- \| --- | not modelled | 100.0 | 21 | **Fold:** alpha/beta-Hydrolases **Superfamily:** alpha/beta-Hydrolases **Family:** Ccg1/TafII250-interacting factor B (Cib) **PDB entry:** PDBe RCSB PDBj |
| 119 | c7a6gA\_ | --- \| --- \| --- | not modelled | 100.0 | 15 |


 (**PDB header:**hydrolase **Chain:** A: **PDB Molecule:**putative proline iminopeptidase; **PDBTitle:** structural characterization of l-proline amide hydrolase from2 pseudomonas syringae **PDB Entry:** PDBe RCSB PDBj) |
| 120 | c6avyA\_ | --- \| --- \| --- | not modelled | 99.9 | 18 |


 (**PDB header:**hydrolase **Chain:** A: **PDB Molecule:**acyl-protein thioesterase 2; **PDBTitle:** crystal structure of zea mays acyl-protein thioesterase 2 **PDB Entry:** PDBe RCSB PDBj) |


|  |  |
| --- | --- |
| Binding site prediction |  |

Automated 3DLigandsite submission is temporarily suspended due to server load. However you may submit models directly to 3DLigandSite HERE

|  |  |
| --- | --- |
| Transmembrane helix prediction |  |

Transmembrane helices have been predicted in your sequence to adopt the topology shown below

---

Phyre is now FREE for commercial users!

All images and data generated by Phyre2 are free to use in any
publication with acknowledgement

**Please cite:** The Phyre2 web portal for protein modeling, prediction and analysis.

Kelley LA *et al.*. *Nature Protocols*
10, 845-858 (2015)[pdf]
[Citation
link]

If you use the binding site
predictions from 3DLigandSite, **please also cite:**

3DLigandSite: predicting ligand-binding sites using similar structures.

Wass MN, Kelley LA and Sternberg
MJ *Nucleic Acids Research* 38, W469-73 (2010) [PubMed]

© Structural Bioinformatics Group

Imperial College London

Lawrence
Kelley, Michael Sternberg

Disclaimer

Terms and Conditions

|  |
| --- |
| **Component software** |
| Template detection: HHpred 1.51 |
| Secondary structure prediction: Psi-pred 2.5 |
| Disorder prediction: Disopred 2.4 |
| Transmembrane prediction: Memsat\_SVM |
| Multi-template modelling and *ab initio*: Poing 1.0 |
